# Cholesterol Sequestration from Caveolae/Lipid Rafts Enhances Cationic Liposome-Mediated Nucleic Acid Delivery into Endothelial Cells

**DOI:** 10.3390/molecules26154626

**Published:** 2021-07-30

**Authors:** Santhosh Chandar Maddila, Chandrashekhar Voshavar, Porkizhi Arjunan, Rashmi Prakash Chowath, Hari Krishna Reddy Rachamalla, Balaji Balakrishnan, Poonkuzhali Balasubramanian, Rajkumar Banerjee, Srujan Marepally

**Affiliations:** 1 Centre for Stem Cell Research (CSCR) (A Unit of inStem, Bengaluru), Christian Medical College Campus, Bagayam, Vellore 632002, TN, India; santhoshchandar2005@gmail.com (S.C.M.); porkizhi.a@cmcvellore.ac.in (P.A.); rashmi.prakash@cmcvellore.ac.in (R.P.C.); 2 Rutgers Biomedical and Health Sciences Rutgers, RWJMS-Institute for Neurological Therapeutics, The State University of New Jersey, 683 Hoes Lane West, Piscataway, NJ 08854, USA; 3 College of Pharmacy and Pharmaceutical Sciences, Florida A&M University, Tallahassee, FL 32307, USA; chanduadb@gmail.com; 4 Chemical Biology Division, CSIR-Indian Institute of Chemical Technology, Hyderabad 560007, TS, India; rahakrireddy@gmail.com; 5 Department of Integrative Biology, School of Bio Sciences and Technology, Vellore Institute of Technology, Vellore 632014, TN, India; balaji.balakrishnan@vit.ac.in; 6 Department of Hematology, Christian Medical College (CMC), Vellore 632004, TN, India; bpoonkuzhali@cmcvellore.ac.in (P.B.); banerjee@iict.res.in (R.B.)

**Keywords:** cationic lipids, clathrin-mediated endocytosis, caveolae-mediated endocytosis, macropinocytosis, transfection, endothelial cells

## Abstract

Delivering nucleic acids into the endothelium has great potential in treating vascular diseases. However, endothelial cells, which line the vasculature, are considered as sensitive in nature and hard to transfect. Low transfection efficacies in endothelial cells limit their potential therapeutic applications. Towards improving the transfection efficiency, we made an effort to understand the internalization of lipoplexes into the cells, which is the first and most critical step in nucleic acid transfections. In this study, we demonstrated that the transient modulation of caveolae/lipid rafts mediated endocytosis with the cholesterol-sequestrating agents, nystatin, filipin III, and siRNA against Cav-1, which significantly increased the transfection properties of cationic lipid-(2-hydroxy-*N*-methyl-*N*,*N*-bis(2-tetradecanamidoethyl)ethanaminium chloride), namely, amide liposomes in combination with 1,2-Dioleoyl-sn-glycero-3-phosphoethanolamine (DOPE) (AD Liposomes) in liver sinusoidal endothelial cells (SK-Hep1). In particular, nystatin was found to be highly effective with 2–3-fold enhanced transfection efficacy when compared with amide liposomes in combination with Cholesterol (AC), by switching lipoplex internalization predominantly through clathrin-mediated endocytosis and macropinocytosis.

## 1. Introduction

Endothelial cells are responsible for forming new blood vessels and regulating vascular permeability and angiogenesis [1]. Aberrant angiogenesis is found in several pathological conditions including tumor vasculature, cardiovascular diseases, diabetic retinopathy, rheumatoid arthritis, and wound healing [2,3,4,5]. Hence, a considerable amount of interest is generated to target endothelial cells to control the disease pathophysiology. Nucleic acid delivery has great potential as a therapeutic strategy and for probing biological mechanisms in vascular diseases [6,7]. However, poor transfection efficiencies in endothelial cells limit their applications [8]. The first and most critical barrier of efficient nucleic acid transfections is intracellular delivery/cellular uptake. Endocytosis plays an essential role in internalizing various biomolecules including proteins and nucleic acids [9]. Therefore, it is important to probe the internalization of lipoplexes and understand the possible mechanisms to maximize the transfection efficiencies. Prior findings, including our own, demonstrated the role of hydrophobic chain asymmetry in the cationic liposomes in modulating the intracellular delivery of the lipoplexes through interacting with lipids of plasma-membrane [10,11]. Understanding the endocytosis process is critical for the efficient delivery of lipoplexes. Predominantly, lipoplexes enter the cell through either clathrin-mediated endocytosis (CME) or clathrin-independent endocytosis (CIE) [9,12]. Earlier studies demonstrated that CME is the efficient endocytic pathway for delivering therapeutic molecules [13,14]. CIE is primarily achieved through caveolae-mediated endocytosis/lipid rafts or macropinocytosis [15]. 

Herein, we report a systematic study on the modulation of liposome-mediated nucleic acid transfection efficacies by inhibition of a caveolae-mediated endocytic pathway, and by diverting the route of lipoplex internalization via clathrin and macropinocytosis. In this study, we employed our previously reported transfection-efficient cationic amphiphile, 2-hydroxy-*N*-methyl-*N*,*N*-bis(2-tetradecanamidoethyl) ethanaminium chloride (Amide Lipid), reported as Lipid 3 in the cited reference [16]. We employed this in combination with co-lipids including DOPE (1,2-Dioleoyl-sn-glycero-3-phosphoethanolamine) and cholesterol at 1:1 mole ratio to form two liposomal formulations, AD (Amide Lipid: DOPE) and AC (Amide Lipid: Cholesterol) respectively, to probe the possible role of the endocytic pathways in modulating their transfection efficacies. A dual approach involving both chemical inhibitors and siRNA was employed to block caveolae-mediated lipoplex cellular uptake and evaluated the effect of the caveolae pit blockade in altering the transfection efficacies in representative endothelial cells (SK-HEP1 cells). Further, the effect on lipoplex internalization and its transfection efficacies in SK-HEP1 cells was assessed under similar inhibitory conditions on clathrin-mediated endocytosis and macropinocytosis. 

## 2. Results

**Figure 1:**
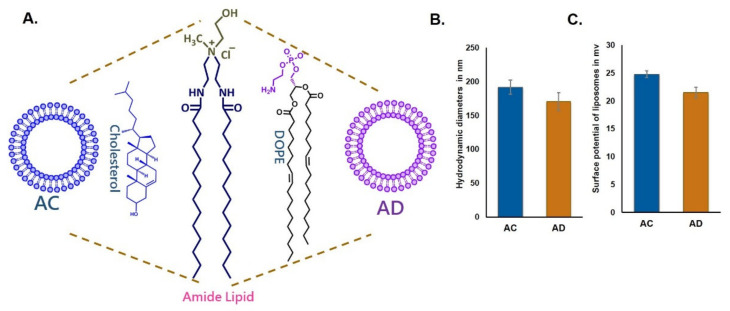
Schematic representation of lipid structures and liposomes of AC and AD (**A**). Particle sizes of liposomes AC and AD (**B**). Zeta potentials of liposomes AC and AD (**C**).

Towards evaluating the influence of cholesterol sequestration on the endocytosis of the liposomes, we prepared two cationic liposomes using the same cationic amide lipid with two different co-lipids, DOPE (AD) and cholesterol (AC) (Figure 1A). Particle sizes were in the range of 170 nm to 200 nm and surface potentials were +21 and +25 mV for both AD and AC liposomes, indicating that the physical characteristics of the liposomes were similar. 

**Figure 2:**
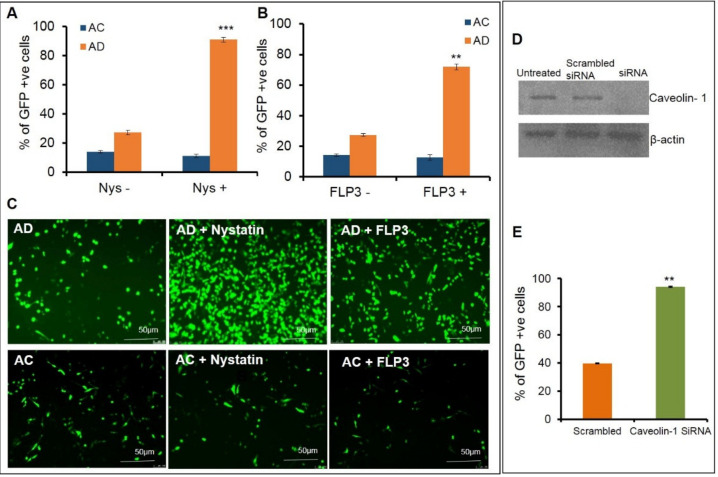
Effect of Caveolae Inhibitors/Blockers on Transfection activity. % of GFP-positive cells assessed by Flow cytometric analysis in nystatin pretreated SK-HEP1 cells (**A**) and filipin III pretreated SK-HEP1 cells (**B**). Representative fluorescence micrographic images for cells transfected using AD (**C** upper panel) and AC (**C** lower panel). Knockdown of Cav-1 with siRNA and transfection activity (**D**,**E**). Caveolin-1 knockdown efficiency demonstrated with Western blot (**D**). Transfection activity (GFP expression) with AD liposomes after knockdown of Caveolin-1 in SK-HEP1 cells assessed by Flow cytometric analysis (**E**), ** *p* < 0.01; *** *p* < 0.001.

Further transfections were carried out using AC and AD liposomes to pretreat endothelial cells with the caveolae blockers, nystatin and filipin III, at varying concentrations. Nystatin was pretreated at different concentrations ranging between 10–50 µg/mL (Supplementary Figure S1). A significant increase, (approximately a ~3–4-fold enhancement) in the transfection efficiencies was observed for AD liposomes at 20 µg/mL concentration in the transfection efficiencies (Figure 2A,C upper panel), whereas the AC liposomes did not affect the transfection properties (Figure 2A,C lower panel). To further confirm these results, cells were pretreated with another caveolae blocker, filipin III, at varying concentrations of 1 µg, 2.5 µg, 5 µg, 7.5 µg and 10 µg (Supplementary Figure S1) followed by transfection with AD. A significant increase in the transfection efficiency was observed, more predominantly at a concentration of 7.5 μg/mL. We observed an approximate increase of 50% in transfection (Figure 2B,C upper panel), whereas the AC liposomes did not show any significant change in the transfection properties (Figure 2B,C lower panel). To further confirm the phenomenon that caveolae blocking has an effect on transfections at the molecular level, we used siRNA against Cav-1. Prior to the transfections, Caveolin-1 was knocked down in SK-Hep1 cells and confirmed with a Western blot analysis. Transfections were performed with AD liposomes in both the Cav-1 knockdown and normal endothelial cells. Approximately, an increase of 50% in transfection efficiencies was observed in the Cav-1 knockdown endothelial cells, when compared to normal endothelial cells. From these findings, we can ascertain that blocking the endocytic entry of lipoplexes through caveolae enhances transfection efficiency (Figure 2D,E).

**Figure 3:**
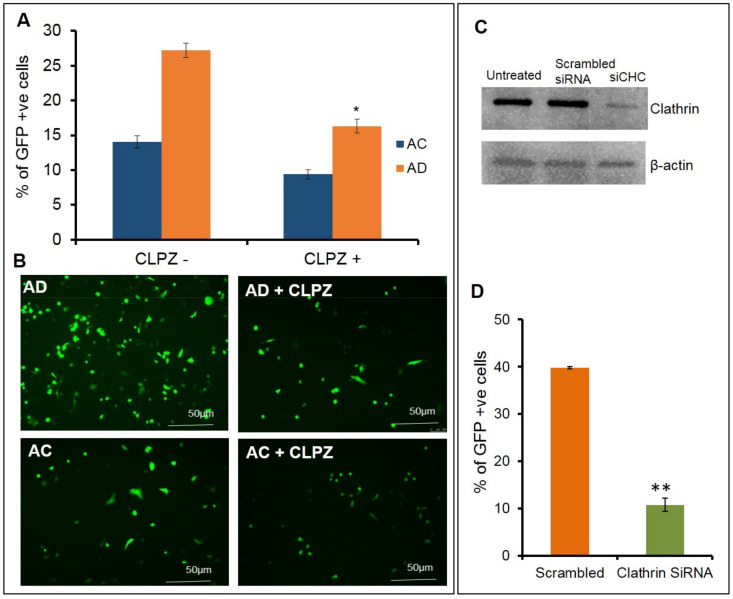
Effect of clathrin inhibitors/blockers on transfection activity. Transfection activity in chlorpromazine (CLPZ) pretreated SK-Hep1 cells (**A**,**B**). % of GFP-positive cells assessed by Flow cytometric analysis (**A**). Representative fluorescence micrographic images of cells transfected using AD (**B** upper panel) and AC (**B** lower panel). Clathrin knockdown efficiency demonstrated with Western blot (**C**). Transfection activity (GFP expression) after knockdown of clathrinid in SK-Hep1 cells assessed by Flow cytometric analysis (**D**). * *p* < 0.05; ** *p* < 0.01.

In order to understand the effect of clathrin blocking in altering the cellular uptake of lipoplexes, next we performed the same experiments with the clathrin blocker, chlorpromazine. Upon pretreating the cells with varying concentrations of chlorpromazine at 0.5 µg, 1 µg, 2 µg, 4 µg, and 8 µg/mL (Supplementary Figure S2), followed by transfection with AD, a decrease in the transfection efficiency was observed. At a 2.5 µg/mL concentration, an approximate decrease of 40% was observed in the transfection (Figure 3A,B upper panel) with AD liposomes and a decrease of 20% was observed in the transfection with AC liposomes. Overall, AD showed a 70% superior transfection than AC. To further confirm the phenomenon that blocking clathrin-mediated endocytosis has an effect on transfections at the molecular level, we used siRNA against the clathrin-heavy chain. Prior to the transfections, clathrin was knocked down in SK-Hep1 cells and confirmed with a Western blot analysis. Transfections were performed with AD liposomes in both clathrin knockdowns and normal endothelial cells. An approximate decrease of 60% in the transfections was observed in the clathrin-knockdown endothelial cells when compared to the normal endothelial cells (Figure 3C,D). These findings revealed that clathrin-mediated endocytosis is the primary pathway for cytosolic entry of lipoplexes, in particular for AD liposomes in endothelial cells.

**Figure 4:**
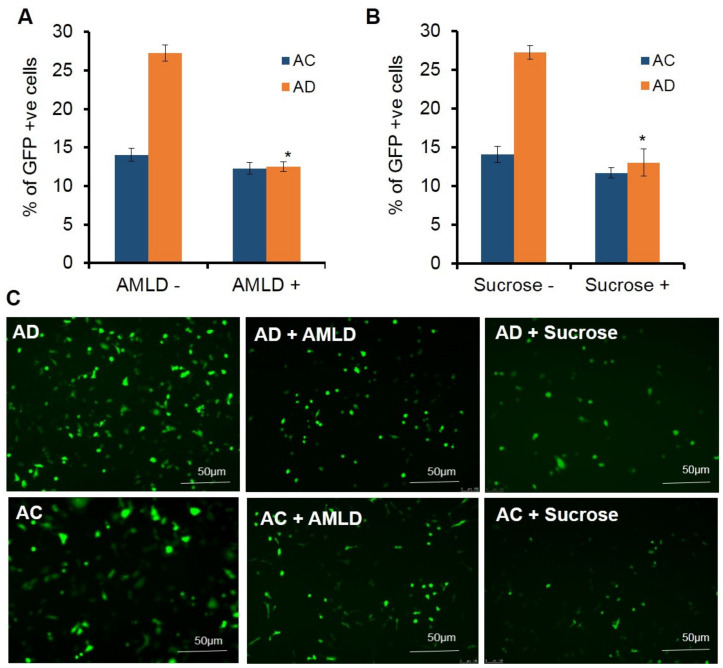
Effect of macropinocytosis inhibitors/blockers on transfection activity. % of GFP-positive cells assessed by Flow cytometric analysis in amiloride (AMLD) pretreated SK-Hep1 cells (**A**). % of GFP-positive cells assessed by Flow cytometric analysis in sucrose pretreated SK-Hep1 cells (**B**). Representative fluorescence micrographic images for cells transfected using AD with and without inhibitors (**C** upper panel). AC with and without inhibitors (**C** lower panel). * *p* < 0.05.

Next, we studied the influence of macropinocytosis in the endocytosis of liposomes in endothelial cells. For this, the cells were pretreated with macropinocytosis blockers, sucrose and amiloride. Upon pretreating the cells with varying concentrations of amiloride (0.1 µg, 0.5 µg, 1 µg, 2 µg, and 4 µg) (Supplementary Figure S3) followed by transfection with AD liposomes, a 2-fold decrease in the transfection efficiency was observed at 4 µg/mL of amiloride (Figure 4A), whereas there was no significant reduction in the transfection efficiencies of AC liposomes. (Figure 4A). Further, to confirm these results, the cells were pretreated with varying concentrations of sucrose (5 µg, 10 µg, 15 µg, 25 µg, and 50 µg/mL) (Supplementary Figure S3) followed by transfection with AD and AC lipoplexes. More than a 2-fold decrease in transfections was observed at a concentration of 25 µg/mL (Figure 4B), whereas the effect of transfections with AC lipoplexes on pretreatment with 25 µg/mL of sucrose was not significant (Figure 4B).

**Figure 5:**
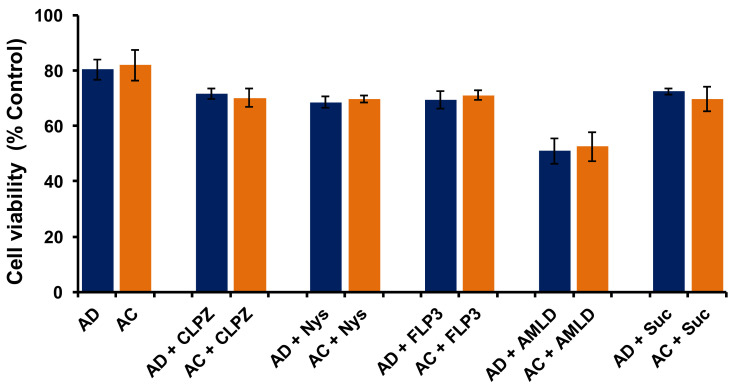
Cell viabilities of SK-Hep1 cells following transfection with AD and AC liposomes in the presence and absence of endocytosis inhibitors.

In order to understand whether cytotoxicities of AD and AC liposomes play any role in determining their transfection properties in SK-Hep1 cells, we performed an MTT-based cell viability assay. Results showed ~80% cell viabilities for both AD and AC liposomes, indicating no significant difference in the cytotoxicity profiles of both liposomes (Figure 5). These findings clearly demonstrate that the poor transfection properties of AC liposomes, when compared to AD liposomes, were not due to cytotoxicity and confirm that the cytotoxicity of individual liposomes has no role in influencing their transfection efficacies. (Figure 5). Further, we performed a cell-viability experiment in the presence of all the endosomal blockers used in the present study at their active concentrations for AD and AC liposomes. All of the blockers except Ameloride demonstrated more than 70% viabilities while Ameloride showed ~60% cell viability (Figure 5). However, there was no significant difference in the cell viabilities of AD and AC liposomes in the presence of each endosomal blocker. These results indicate that the application of endosomal blockers for switching the endocytosis pathways in liposomal transfections is safe. 

Overall, the liposomes with DOPE (AD) showed better transfection efficiencies when compared to cholesterol-doped liposomes (AC) in endothelial cells. Transfection experiments with different endocytic blockers revealed that the AD lipoplexes predominantly enter through clathrin and micropinocytosis pathways in endothelial cells. Blocking caveolae-mediated endocytosis significantly improved the transfection efficiencies of the liposomes which do not have cholesterol as a co-lipid (AD), when compared to liposomes containing cholesterol as a co-lipid (AC).

## 3. Discussion

Owing to the poor transfection efficiencies with the currently available transfection reagents in endothelial cells, gene therapy applications in vascular diseases are limited [17]. The first and most critical step of gene delivery is the cellular uptake that proceeds through the endocytosis pathway [13,18]. The cellular entry of lipoplexes or polyplexes is predominantly through either caveolae-mediated endocytosis, clathrin-mediated endocytosis, or macropinocytosis. Caveolae pits are 50 to 100 nm plasma-membrane invaginations in the cell surface and are abundant in endothelial cells [19]. These are responsible for the regulation of endothelial vesicular trafficking and signal transduction [20]; however, these uptake regulatory mechanisms are complex in nature. Understanding the endocytic mechanisms in endothelial cells helps in developing effective delivery systems and routes which ultimately yield increased transfection efficacies. Several mechanistic investigations have been carried out previously using various pharmacological inhibitors that are routinely employed to investigate the endocytic pathways [21]. These inhibitors are chemical (small molecules and macrolides), physical (temperature, pressure, and electric field), and biological (siRNA/shRNA) in nature [22,23,24]. In this study, we used nystatin and filipin III as caveolae inhibitors; chlorpromazine as a clathrin inhibitor; and sucrose and amiloride as macropinocytosis inhibitors. In the present study, we used our previously reported transfection efficient, the amide lipid, for delivering nucleic acids [16,25]. Towards understanding the influence of co-lipids on the cellular uptake of lipoplexes in the presence of endocytic blockers, we used cholesterol and DOPE in combination with the amide lipid. Firstly, we evaluated the sizes and surface potentials of liposomes, AC and AD. Both of the liposomes’ physicochemical characteristics, including particle size and surface potential, were found to be in a similar range. Further, we confirmed that these liposomes were found to be safe for transfection studies with cell viability assay. Both these studies showed change of co-lipid did not influence physicochemical properties and cytotoxicities of AC and AD liposomes. Next, we evaluated transfection properties in the presence of blockers of multiple endocyic pathways including caveolae, clathrin and macropinocytosis. When transfections were performed in presence of caveolae blockers including nystatin and filipin III, a significant increase in transfections was observed with lipoplexes of AD liposomes, whereas lipoplexes of AC liposomes were unchanged. This increase could possibly be due to the cholesterol sequestration from the caveolae pits of the endothelial cells, switching endocytic pathways from caveolae to more transfection favourableClathrin-Mediated Endocytosis. Further, we confirmed the effect of cholesterol sequestration by supplementing cholesterol in AC liposomes) in which transfection efficiencies were unaffected, even after cholesterol sequestration. These findings are in corroboration with the previous reports of protein delivery in endothelial cells [26,27]. Cholesterol sequestration in endothelial cells might be due to the high abundance of caveolae in endothelial cells [28]. We confirmed this by knocking down a caveolin-1 protein with siRNA against Cav-1. Next, we evaluated the effect of this with the clathrin blocker, chlorpromazine, and found that transfection efficiencies of both AC and AD liposomes were retarded. However, the extent of this decrease is more significant in AD when compared to AC, suggesting that AD has favorable clathrin-mediated cellular internalization over AC. We further confirmed this effect by knocking down the clathrin-heavy chain using RNA interference in SK-Hep1 cells and performed the transfection with lipoplexes of AD liposomes. Towards probing the effect of macropinocytosis, we performed the transfections with macropinocytosis inhibitors including amiloride and sucrose. Transfections of lipoplexes of AD liposomes showed significant reduction whereas lipoplexes of AC liposomes remained almost the same in the presence of both inhibitors. Overall, our study demonstrated that AD liposomes showed enhanced transfection ability when compared to AC liposomes and that switching endocytic entry from caveolae to clathrin by cholesterol sequestration in endocytic cells significantly improved the transfection properties of AD liposomes. 

## 4. Materials and Methods

### 4.1. Cell Culture

SK-HEP1 (Human Hepatic Adenocarcinoma Cell Line) is an immortal, human cell line of endothelial origin, derived from the ascitic fluid of a patient with adenocarcinoma of the liver, obtained from ATCC, USA [29]. Cell lines were maintained in Eagle’s Minimum Essential Medium Eagle (EMEM) (Gibco^TM^) and supplemented with 10% fetal bovine serum (FBS) from Gibco^TM^ (ThermoFischer Scientific, USA) and an antibiotic mixture comprising of penicillin (5000 U/mL) and streptomycin (0.1 mg/mL) from Gibco^TM^ (ThermoFischer Scientific, USA) and cells were subsequently incubated at 37 °C and 5% CO_2._ The growth media were replaced every 2 days and the cells were sub-cultured by treating the cells with 0.25% trypsin-EDTA (Gibco^TM^) when the cells were 80–90% confluent. Unless stated, other common chemicals were purchased from local suppliers in India.

### 4.2. Preparation of Cationic Lipid Nanocarriers and Plasmid 

The 1 mM liposomes of cationic amide lipid (2-hydroxy-*N*-methyl-*N*,*N*-bis(2-tetradecanamidoethyl)ethanaminium chloride) with DOPE (1,2-Dioleoyl-sn-glycero-3-phosphoethanolamine, TCI Chemicals, Japan)/cholesterol (HiMedia, India) was prepared by a solvent-evaporation method and the lipid film was kept for drying under high vacuum for 1 h. One milliliter of sterile deionized water was added to the vacuum-dried lipid films and the mixtures were allowed to stay overnight. The vials were then vortexed for 2–3 min at room temperature to produce multilamellar vesicles (MLVs). MLVs were then sonicated initially in a water bath followed by probe sonication using a Branson 450 sonifier, USA at 100% duty cycle and 25 W output power to produce small unilamellar vesicles (SUVs). eGFP plasmid amplified in DH5α-strain of *Escherichia coli* and the purity of plasmid was checked by A260/A280 ratio (around 1.9). 

### 4.3. Zeta Potential (ξ) and Size Measurements of Liposomes 

The sizes and the zeta potentials (surface charges) of liposomes were measured by photon-correlation spectroscopy and electrophoretic mobility using a particle size analyzer (Anton Paar Litesizer 100, Germany). The sizes and potentials of liposomes were measured in deionised water with a sample refractive index of 1.59 and a viscosity of 0.89. The diameters of liposomes and lipoplexes were calculated by using the automatic mode. The zeta potential was measured using the following parameters: viscosity, 0.89 cP; dielectric constant, 79; temperature, 25 °C; F(Ka), 1.50 (Smoluchowski); The potentials were calculated by using the Smoluchowski approximation. 

### 4.4. Transfection Assay

50,000 (SK-Hep1) cells were seeded per well in a 24-well plate, after 18–24 h, 0.5 μg of plasmid DNA was complexed with varying amounts of lipids (0.9–7.2 nmol) in plain MEM medium (total volume was made up to 100 μL) and incubated for 30 min at room temperature. The lipid: DNA (+/–) charge ratio was standardized in the lab and the 4:1 ratio was found to render optimum and consistent results. The transfection efficiency was measured after 36–48 h of transfection. Leica Fluorescence microscopy and FACS analysis were used to record the transfection efficiencies.

### 4.5. Transfection in Presence of Various Endocytic Blockers

Transfections were performed by pretreating the cells with different types of endocytic blockers to study the mechanism of uptake. We probed into different endocytic pathways by using inhibitors for specific pathways. Caveolae blockers: nystatin and filipin 3; Clathrin blockers: chlorpromazine; Macropinocytosis blockers: amiloride and sucrose. These blockers are used at different concentrations as shown below. Concentrations of nystatin (10 µg, 20 µg, 30 µg, 40 µg and 50 µg). Concentrations of Filipin-3 (1 µg, 2.5 µg, 5 µg, 7.5 µg and 10 µg). Concentrations of chlorpromazine (0.5 µg, 1 µg, 2 µg, 4 µg and 8 µg). Concentrations of amiloride (0.1 µg, 0.5 µg, 1 µg, 2 µg, 4 µg). Concentrations of sucrose (5 µg, 10 µg, 15 µg, 25 µg and 50 µg). Cells were pretreated with inhibitors for 30 min and then cells were briefly washed; then the lipoplexes were added with an active charge ratio of 4:1 to the cells. (Shown in Supplementary).

### 4.6. Fluorescence Microscopy 

To detect the transfection efficiency using lipids, the cells were treated with varying lipid:pDNA charge ratios. The fluorescence was monitored by using Leica fluorescence microscope, (Leica CTR 6000, Germany) after every 36–48 h of transfection. The fluorescence intensity was analyzed by calculating mean fluorescent intensity using LAS AF software (AF6000 DFCV3.6).

### 4.7. Flow Cytometry

For the detection of eGFP-positive cells, (10^4^ cells/well) cells complexed with lipoplexes were harvested after 48 h of transfection and resuspended in 1xPBS. Analysis was carried out on a FACS BD Celesta Flow cytometry, USA. shRNA mediated knockdown: Knockdown of clathrin-heavy chain and caveolin-1 protein was achieved using siRNA, obtained from RiboBio Co., Ltd. China It was introduced into cells in lipid-mediated transfection, where cells took in non-covalent complexes between nucleic acid and a lipid reagent by endocytosis. The efficiency of the knockdown was verified by Western blot. The cells were further used to check for transfection efficiency using lipids to see the efficacy of transfection in normal cells and cells with knockdown for a specific protein.

### 4.8. Western Blot

Semi-dry Western blot was performed according to Towbin et al. (1979) [30]. The protein which was isolated from the cell was subjected to SDS-PAGE. The completed gel electrogram was blotted onto a sheet of PVDF (Polyvinyl difluoride) membrane that had been activated by briefly soaking it in methanol, then quickly transferring it into a transfer buffer. The transfer was set up in a sandwich model which is made up of a cathode graphite plate onto which a sponge, filter papers and the gel electrogram pre-soaked in cathode buffer was laid. The PVDF membrane was laid onto the gel electrogram followed by filter paper and a sponge pre-soaked in transfer buffer. Finally, an anode graphite plate was laid on top of the setup to complete the transfer cassette. The transfer cassette was placed in the blotting chamber. The transfer process was carried out at 350–400 mA for 60 min. The PVDF membrane binds proteins strongly and unspecifically. The excess adsorption sites on the PVDF were blocked using 5% BSA (Bovine serum albumin) in TBS (Tris-buffered saline) buffer so as to prevent non-specific adsorption of antibodies. The membrane was rinsed 3 times for 10 min each with TBST (TBS+Tween 20) and incubated with the primary antibody, the antibody for the protein of interest (anti-clathrin-heavy chain BD 610500) or Caveolin-1 BD 611339) for 1 h. After washing away the unbound primary anti-body with TBST (3 times for 10 min each), the blot was incubated for 1 h with a secondary antibody directed against the primary antibody to which the protein of interest had been covalently linked. After washing away, the unbound secondary antibody (3 times for 10 min each) with TBST, immunostaining of the membrane was performed by exposing the membrane to an enhanced chemiluminescence mixture (ELS) for 1 min. The blot was imaged using Fluorchem E from Cell Biosciences. 

### 4.9. Cell Viability Assay

The cell viability of SK-Hep1 cells in transfection experiment with AD and AC liposomes was estimated using MTT assay; MTT is 3-(4,5-dimethylthiazol-2-yl)-2,5-diphenyltetrazolium bromide which reduces to insoluble Formosan. The cells were seeded in 96 well plates at density of 10^4^ cells/well and incubated in 5% CO_2_ incubator at 37 °C overnight. The cells were treated with lipoplexes of AD and AC liposomes in presence and absence of endosomal blockers and incubated for 24 h. The cells were then incubated with 5 mg/mL of MTT for 2 h at 37 °C. Then, 100 µL of Dimethylsulphoxide (DMSO) was added to neutralize the reaction. After 15 min absorbance, the absorbance was recorded at 550 nm using ELISA plate reader (Thermo Multiskan EX 355, USA). Results were expressed as percent viability of cells = [treated wells − background/control wells − background] × 100.

### 4.10. Statistical Analysis 

The data were shown as the mean ± standard deviation for at least three replicates. The multiple groups were compared using a one-way analysis of variance (ANOVA) and between two groups by student’s *t*-test analysis using GraphPad Prism Version 5.01 for Windows (GraphPad Software Inc.) 

## 5. Conclusions

Despite advances in nucleic acid delivery, transfection efficiencies of liposomal vectors in targeting the vasculature is still considered to be sub-optimal for making this technology a viable therapeutic option. Here, we reported that sequestering cholesterol in caveolae pits of endothelial cells with nystatin, switches lipoplex internalization to pro-transfection routes, i.e., clathrin-mediated endocytosis and macropinocytosis in endothelial cells (SK-Hep1). In conclusion, our findings convincingly demonstrated that the internalization of the cationic lipid-DOPE lipoplexes is mediated by macropinocytosis and negatively regulated by caveolae pits in SK-HEP1 cells. Nystatin sequesters cholesterol from caveolae pits that in turn switches endocytosis of the lipoplexes from the low-efficiency caveolae/lipid rafts to the high-efficiency macropinocytosis, resulting in enhanced lipoplex internalization and transgene expression in the endothelial cells. For the first time, this study demonstrated that cholesterol sequestration leads to an enhanced cellular uptake and efficacy of the lipid-mediated gene delivery and genome editing via regulating distinct endocytic pathways. This approach can be further explored in gene therapy for vascular diseases. 

## Data Availability

Original images of western blot were submitted to Molecules editorial office. Other raw data can be provided on the request.

## Supplementary Materials

The following are available online, Figure S1: Concentration dependent optimization of Caveolae Inhibitors for Endocytosis; Figure S2: Concentration dependent optimization of Clathrin Inhibitors for Endocytosis; Figure S3. Concentration dependent optimization of Macropinocytosis Inhibitors for Endocytosis.

## Sample Availability

Samples of the compounds are not available from the authors.
