# Supplementary material for: Cholesterol Sequestration from Caveolae/Lipid Rafts Enhances Cationic Liposome-Mediated Nucleic Acid Delivery into Endothelial Cells
Source: Molecules. 2021 Jul 30;26(15):4626. doi: 10.3390/molecules26154626 (PMC8346983; doi:10.3390/molecules26154626)
Supplement: Supplementary file 1 [file molecules-26-04626-s001.zip › molecules-1267460-supplementary.pdf]

molecules

MDPI

Supplementary Information

Transfections in presence of Caveolae blockers

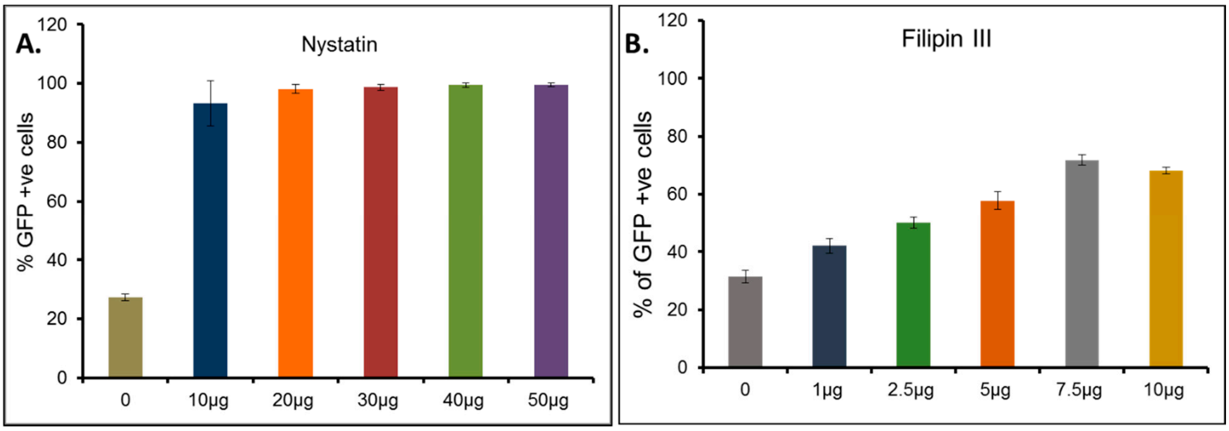

Figure S1. Concentration dependent optimization of Caveolae Inhibitors for Endocytosis. Transfection efficiencies of AD in Nystatin pre-treated SK-HEP-1 cells (A), Filipin III pre-treated SK-HEP-1 cells (B). % of GFP positive cells assessed by Flow cytometric analysis in Nystatin pre-treated SK-HEP-1 cells (A), Filipin III pre-treated SK-HEP-1 cells (B). Cells were pretreated with chemical inhibitors (Nystatin & Filipin-III) to block caveolae mediated endocytosis pathway. Transfections were performed as described in Materials & Methods section.

Transfections in presence of Clathrin blockers

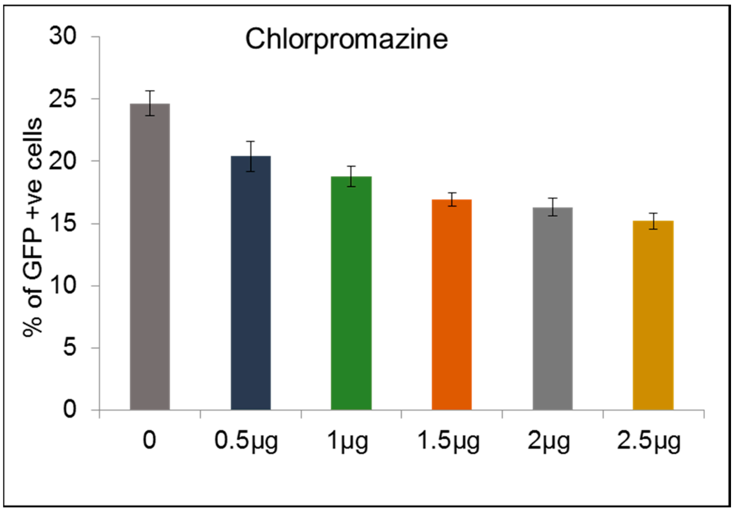

Figure S2. Concentration dependent optimization of Clathrin Inhibitors for Endocytosis. Transfection activity of AD in Chlorpromazine pre-treated SK-HEP-1 cells. % of GFP positive cells assessed by Flow cytometric analysis in Chlorpromazine pre-treated SK-HEP-1 cells. (**A**).

molecules MDPI

Transfections in presence of Macropinocytosis blockers

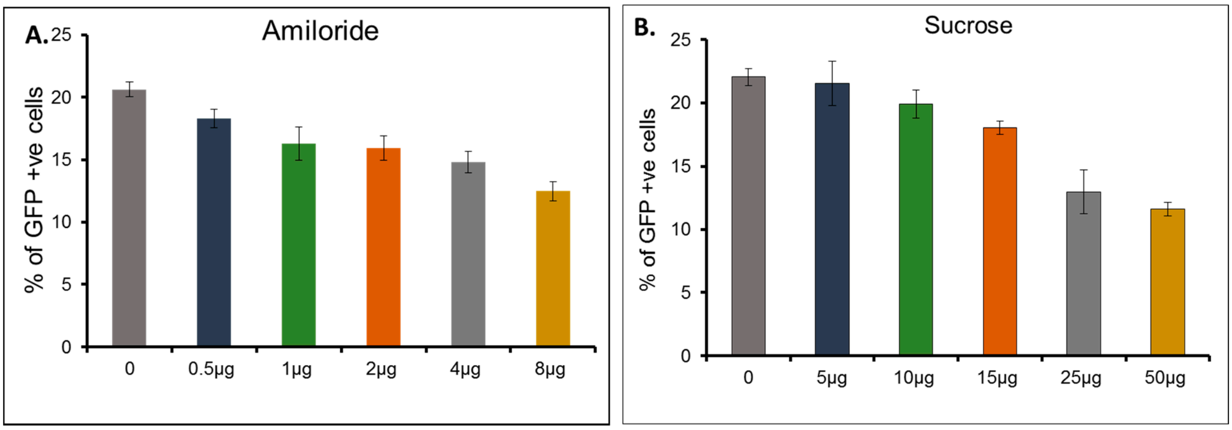

Figure S3. Concentration dependent optimization of Macropinocytosis Inhibitors for Endocytosis Transfection efficiencies of AD in Amiloride pre-treated SK-HEP-1 cells (A), Sucrose III pre-treated SK-HEP-1 cells (B). % of GFP positive cells assessed by Flow cytometric analysis in Amiloride pre-treated SK-HEP-1 cells (A), Sucrose pre-treated SK-HEP-1 cells (B). Cells were pretreated with chemical inhibitors (Amiloride & Sucrose) to block Macropinocytosis mediated endocytosis pathway. Transfections were performed as described in Materials & Methods section.
